# Uniportal non-coaxial endoscopic posterior cervical discectomy with annular suture repair for C6/C7 disc herniation: a case report

**DOI:** 10.3389/fsurg.2025.1733374

**Published:** 2026-01-22

**Authors:** Yaoyu Xiang, Xin Zhang, Fei Sun, Xianguang Yang, Xidan Hu, Jing Yang, Weiqing Ge, Tao Zhou, Yixiao Wang, En Song

**Affiliations:** 1 Department of Sports Medicine, First Affiliated Hospital of Kunming Medical University, Kunming, Yunnan, China; 2 Department of Orthopedics, Kunming Orthopedics Hospital, Kunming, Yunnan, China; 3 Department of Orthopedics, Traditional Chinese Medicine Hospital of Luliang County, Qujing, Yunnan, China; 4 Clinical Pharmacy Center, First Affiliated Hospital of Kunming Medical University, Kunming, Yunnan, China

**Keywords:** annular repair, cervical radiculopathy, endoscopic discectomy, ligamentum flavum preservation, minimally invasive spine surgery, non-coaxial endoscopy, uniportal spinal endoscopy

## Abstract

**Background:**

Cervical disc herniation with radiculopathy is a common cause of neck and arm pain. While anterior cervical discectomy and fusion (ACDF) remains the standard treatment, it sacrifices motion and may cause adjacent segment degeneration. Uniportal non-coaxial spinal endoscopic surgery (UNSES) offers a motion-preserving alternative. This case presents the first application of UNSES with endoscopic annular suture repair and ligamentum flavum suspension in the cervical spine, demonstrating its technical feasibility.

**Case presentation:**

A 54-year-old male presented with progressive neck and right right arm pain, numbness and triceps weakness, due to right paracentral C6/C7 disc herniation compressing the C7 nerve root. The patient underwent full-endoscopic posterior cervical discectomy using a uniportal non-coaxial endoscopic system, with ligamentum flavum preservation via suture suspension. The annular defect was repaired intraoperatively using an endoscopic annular suture device under direct visualization. Postoperative imaging confirmed complete neural decompression and successful annular closure without residual disc or dural compromise. Postoperative MRI confirmed complete decompression and annular closure. At 3 months, visual analog scale (VAS) improved from 7 to 1, the Japanese Orthopaedic Association (JOA) score increased from 13 to 16, and the Neck Disability Index (NDI) decreased from 42% to 14%, with no recurrence or instability.

**Conclusions:**

UNSES combined with annular suture repair enables safe, motion-preserving decompression for cervical disc herniation. This novel approach may enhance biomechanical integrity, reduce recurrence, and represent a minimally invasive alternative to fusion in selected patients.

## 1 Introduction

Cervical radiculopathy is a common neurological condition characterized by pain radiating from the neck to the arm, often accompanied by numbness, sensory deficits, or motor weakness in the affected dermatome (1). The most frequently involved segments are C5/C6 and C6/C7, corresponding to C6 and C7 nerve roots, respectively. Epidemiological studies indicate an annual incidence of approximately 83.2 per 100,000 individuals, peaking in the fifth decade of life (2). The main pathological mechanism is compression or irritation of cervical nerve roots by disc herniation or spondylotic changes, resulting in radicular pain and functional impairment.

Traditional surgical treatments for cervical radiculopathy primarily include anterior cervical discectomy and fusion (ACDF) and posterior cervical foraminotomy (PCF)**.** Although ACDF remains the gold standard for direct decompression of ventral lesions and provides reliable symptom relief, it inevitably causes segmental immobility, leading to adjacent segment degeneration (ASD) and hardware-related complications such as pseudoarthrosis and dysphagia (3). In contrast, posterior open decompression techniques like laminoforaminotomy, preserve motion but often require extensive paraspinal muscle dissection, which can lead to postoperative pain, muscle atrophy, and prolonged recovery periods (4). Both approaches also carry risks of blood loss, infections, and longer hospitalization,which highlight the growing need for less invasive, motion-preserving alternatives (5).

Uniportal Noncoaxial Spinal Endoscopic Surgery (UNSES), also known as Arthroscopic-Assisted Uniportal Spinal Surgery (AUSS), has emerged as a promising solution for precise decompression through a small working portal. Compared to traditional open surgery, UNSES features a single working channel with an independent, non-coaxial endoscope trajectory, allowing greater flexibility in confined anatomical spaces and minimizing collateral tissue injury (6). Originally utilized primarily in the lumbar pathologies, UNSES has demonstrated shorter operative time, less blood loss, and faster recovery than conventional open techniques (7). These advantages suggest that UNSES could be safely extended to cervical applications, achieving adequate neural decompression while preserving stability and motion.

Despite the clinical success of endoscopic decompression in the lumbar spine, the combination of UNSES with annular suture repair in the cervical spine has not been previously reported. The annulus fibrosus plays a critical role in maintaining intervertebral disc integrity and preventing recurrence after discectomy. Incorporating annular closure into cervical endoscopy could theoretically reduce reherniation risk and preserve biomechanical stability (8). Additionally, ligamentum flavum (LF) preservation and suspension during endoscopic exposure may further minimize epidural fibrosis and maintain posterior tension band integrity (9). Therefore, this case report presents the first clinical application of UNSES with endoscopic annular suture repair and LF preservation for C6/C7 disc herniation, highlighting the feasibility, safety, and motion-preserving potential of this novel approach.

## 2 Case presentation

A 54-year-old male presented with a 4-month history of progressive neck pain radiating to the right upper limb, following heavy lifting. Symptoms included numbness and restricted cervical mobility. He initially self-managed using oral celecoxib, shockwave therapy, physiotherapy, and intravenous infusions; however, specific details of the infusions were unavailable, and these treatments provided minimal relief. He denied any history of trauma, cervical spine surgery, or systemic neurological disorders. Due to persistent symptoms and functional impairment, he was referred to our institution for further evaluation, including cervical spine MRI.

The patient presented with limited cervical mobility, particularly in extension and right lateral rotation. Spurling's test elicited radicular pain in the right posterior forearm. Neurological examination demonstrated decreased pinprick sensation over the right posterior forearm and middle finger, reduced triceps strength (MRC grade 4/5), and a diminished triceps reflex—findings indicative of right C7 radiculopathy.

**Figure 1:**
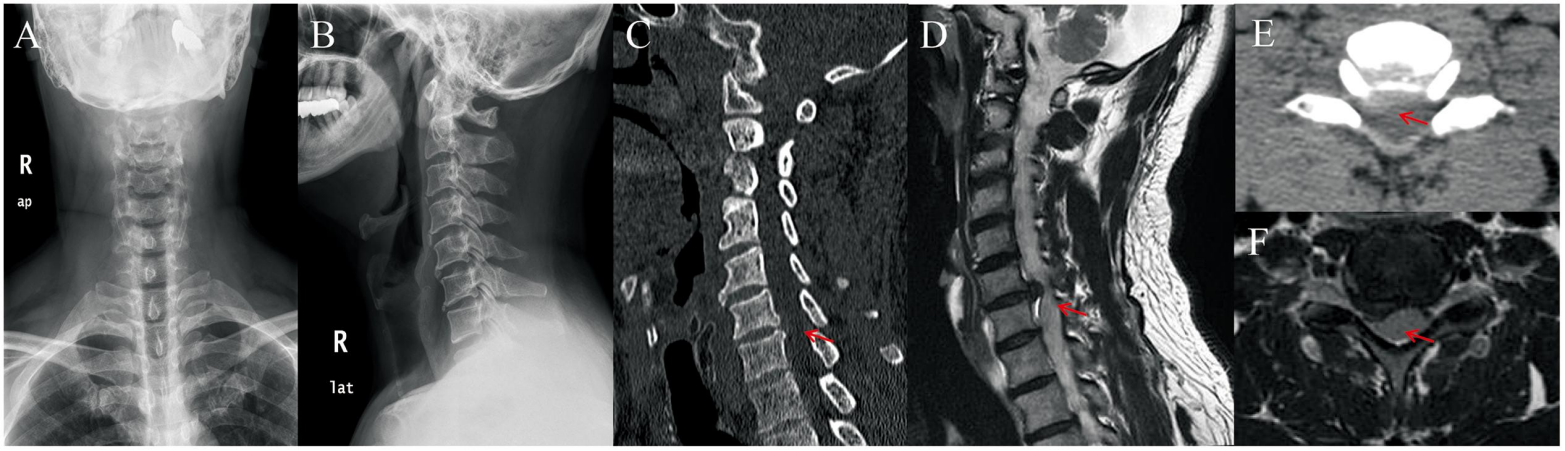
Preoperative imaging of the C6/C7 disc herniation. **(A)** Anteroposterior radiograph showing preserved cervical alignment without scoliosis or deformity; **(B)** lateral radiograph demonstrating normal cervical lordosis and absence of segmental instability; **(C)** sagittal CT reconstruction revealing disc space narrowing and right paracentral osteophyte formation at the C6/C7 level (red arrow); **(D)** sagittal T2-weighted MRI showing a right paracentral C6/C7 disc herniation compressing the exiting C7 nerve root (red arrow); **(E)** axial CT image confirming right-sided disc-osteophyte complex encroaching on the lateral recess (red arrow); **(F)** axial T2-weighted MRI demonstrating focal right C6/C7 disc protrusion compressing the C7 nerve root (red arrow).

Dynamic cervical radiographs demonstrated preserved cervical alignment without evidence of instability (Figures 1A,B). CT scans confirmed disc space narrowing and right-sided marginal osteophyte formation at the C6/C7 level (Figures 1C,E). MRI revealed a right paracentral C6/C7 disc herniation compressing the right C7 nerve root within the lateral recess (Figures 1D,F).

Preoperative assessment yielded a VAS score of 7/10 for right arm pain, a JOA score of 13/17, and a Neck Disability Index of 42%, supporting the diagnosis of right-sided C7 radiculopathy secondary to C6/C7 disc herniation.

## 3 Surgical technique

All procedures were performed under general anesthesia with the patient placed prone on a radiolucent surgical table.

### 3.1 Positioning and localization

A three-step lateral fluoroscopic localization technique was used to accurately identify the target level (C6/C7). The patient's head was supported on a radiolucent horseshoe headrest, providing stable, noninvasive fixation in the prone position. Because the headrest partially obstructs the anteroposterior view, all fluoroscopic confirmations were performed in the lateral projection. First, prior to draping, a Kirschner wire was placed in the lateral neck for preliminary identification of the C6/C7 disc space under lateral fluoroscopy. The skin entry point was marked at the intersection between the disc level and a line connecting the pedicle midpoints of the upper and lower vertebrae on the symptomatic side. After draping, a 10-mL syringe needle was inserted percutaneously at this point and confirmed under lateral fluoroscopy (second localization). A 5-mm skin incision was then made at the confirmed entry site. A primary dilator was advanced toward the junction of the lamina and spinous process until bony resistance was palpated, corresponding to the inferior edge of the C6 lamina. A third lateral fluoroscopic confirmation was performed, and the incision was adjusted upward or downward as needed to approximately 1.5–2 cm to establish the optimal working trajectory.

### 3.2 Laminotomy and exposure

**Figure 2:**
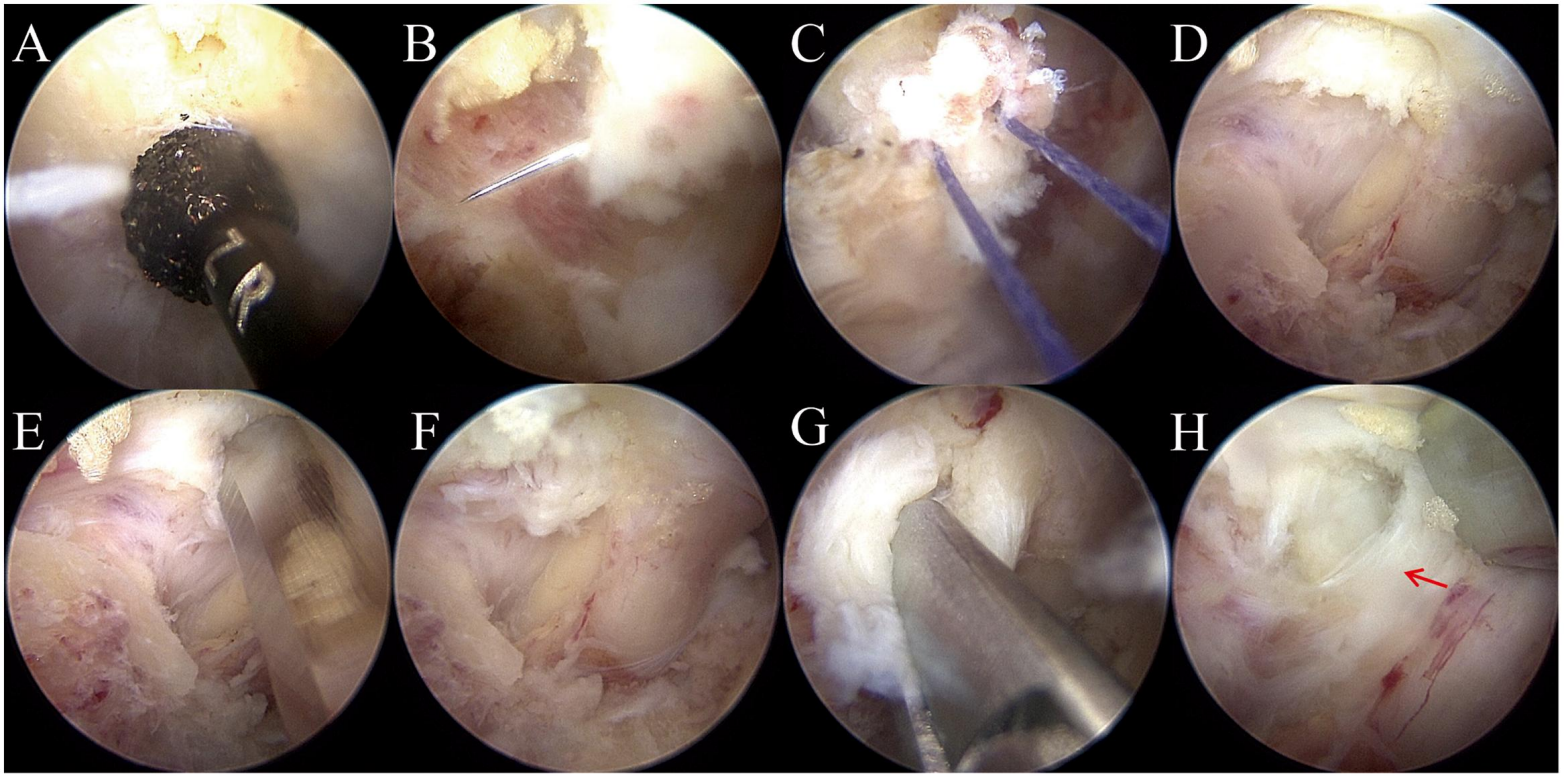
Intraoperative endoscopic views demonstrating key procedural steps of posterior cervical discectomy and annular closure at the C6/C7 level. **(A)** The interlaminar window was created using a high-speed diamond burr to thin the inferior margin of the C6 lamina and the superior margin of the C7 lamina; **(B)** passage of a 4-0 absorbable suture through the lateral margin of the LF near the facet joint using pituitary forceps, establishing a single-stitch suspension pathway; **(C)** suspension of the LF was achieved using a 4-0 absorbable suture passed laterally through the ligament to provide gentle retraction and a clear operative corridor; **(D)** identification of the focal disc bulge located in the axillary zone beneath the right C7 nerve root; **(E)** annulotomy was performed using a surgical blade to expose the extruded nucleus pulposus; **(F)** removal of the herniated disc fragment with pituitary forceps under direct endoscopic visualization; **(G)** final inspection of the decompressed right C7 nerve root confirming complete neural decompression; **(H)** visualization of the medial annular defect (red arrow) adjacent to the dura, marking the site prepared for endoscopic annular repair.

Under endoscopic visualization, the C6/C7 “V-point” was cleared using bipolar plasma radiofrequency ablation. A 4-mm diamond burr was used to perform laminotomy, gradually thinning the inferior edge of the C6 lamina and the superior edge of the C7 lamina until the cranial, caudal, and medial insertions of the LF were visualized (Figure 2A). Bone removal was performed layer by layer with continuous hemostasis to avoid over-penetration of the burr. A 130 ° 2-mm Kerrison rongeur was then used to free the margins of the LF circumferentially. Bipolar radiofrequency was applied to release adhesions and achieve hemostasis along the ventral aspect of the LF, ensuring complete mobilization of the ligament while avoiding inadvertent dural contact.

### 3.3 Ligamentum flavum suspension

Once the LF was fully delineated, suspension was performed to enhance exposure while preserving its structural integrity. A 4-0 absorbable suture with an attached needle (Vicryl™, Ethicon Inc.) was grasped at its midpoint using pituitary forceps and introduced through the working channel. The needle was inserted through the lateral margin of the LF near the facet joint and retrieved externally with another pituitary forceps (Figure 2B), where the ligament is relatively thick and safely distant from the dura. The suture was cut at the needle end, and both free ends were clamped together with a hemostat, which was gently hung outside the surgical field (Figure 2C). The weight of the clamp provided mild tension—approximately equivalent to the weight of a straight surgical instrument—creating a stable lateral retraction that elevated the LF and exposed the dura and nerve root. This maneuver maintained the ligament's continuity and avoided any direct dural traction.

### 3.4 Annular suture repair

**Figure 3:**
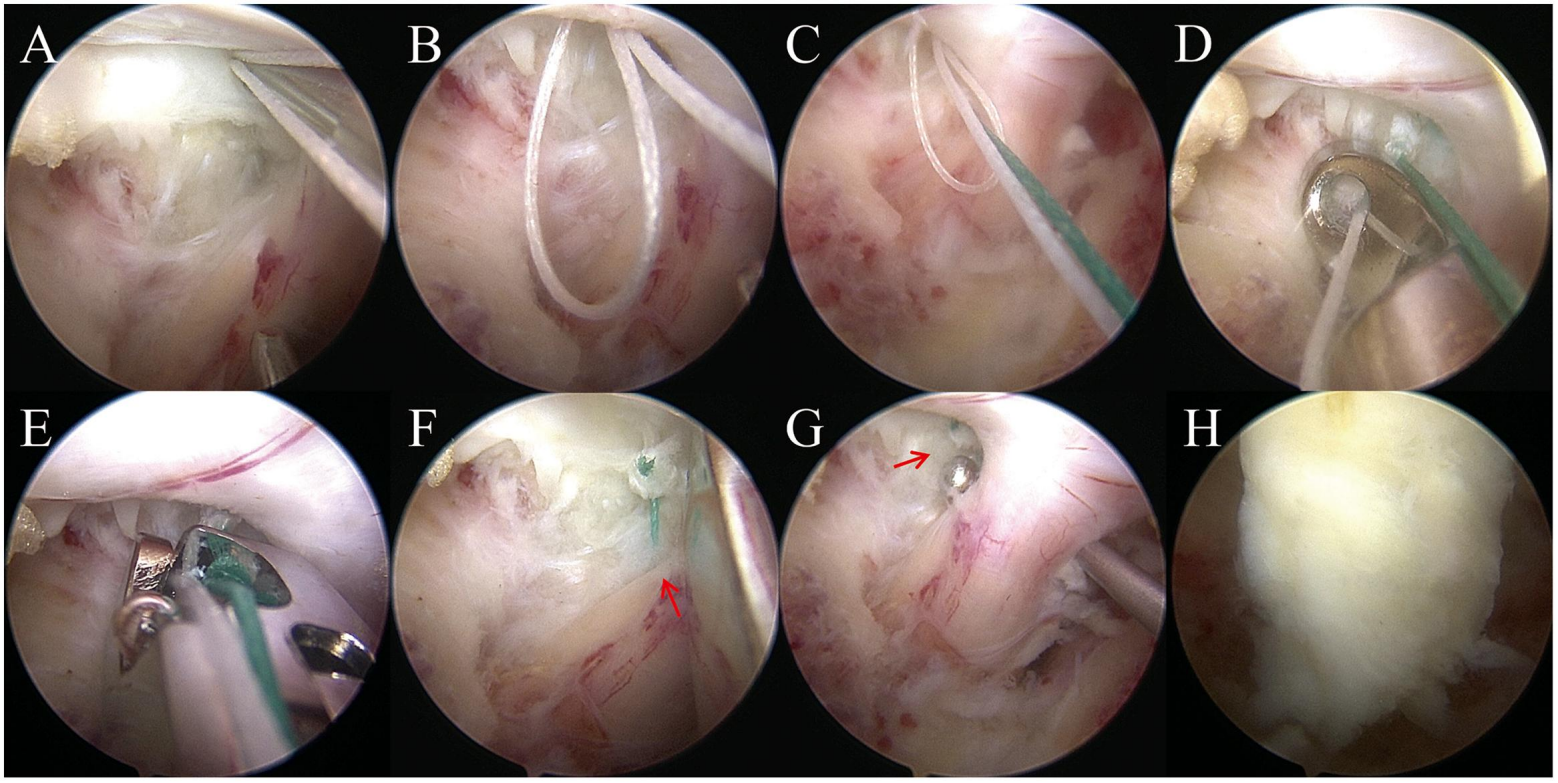
Endoscopic annular repair at the C6/C7 level. **(A)** Selection of the suture entry point approximately 3 mm from the edge of the annular defect, targeting the firm outer annular tissue for secure anchorage. The first (white) needle carrying a self-locking loop suture is advanced through this point; **(B)** retrieval of the first needle after piercing the annular margin, revealing the white self-locking suture loop within the operative field; **(C)** insertion of the second (green) suture through the opposite side of the annular defect and into the white loop, ensuring passage across the fissure under endoscopic guidance; **(D)** Extracanalicular knot tying performed using a knot pusher outside the spinal canal, with the knot positioned away from neural structures; **(E)** cutting of excess suture with endoscopic scissors at the designated trimming site (arrow); **(F)** visualization of the remaining suture tail following trimming (arrow); **(G)** final endoscopic inspection confirming complete neural decompression and extracanalicular knot placement outside the canal (arrow). **(H)** Repositioning of the LF to restore anatomical continuity after completion of annular repair.

The focal disc bulge was identified in the axillary zone, located beneath the right C7 nerve root (Figure 2D). A standard scalpel was used to incise the annulus fibrosus, and the extruded nucleus pulposus was extracted using pituitary forceps (Figures 2E–G). Following decompression, the annular defect—situated medially near the dura—was clearly visualized (Figure 2H). Annular closure was performed using a dedicated endoscopic annular suture system (Annular Suture Device, 2020 Inc., China), which consists of two needles—a white needle carrying a self-locking loop and a green needle carrying a simple suture loop. Several technical points were critical for successful repair in this cervical case. First, owing to the near-vertical orientation of the soft working channel relative to the C6/C7 disc space, combined with adequate bony decompression, LF suspension, soft tissue clearance, and hemostasis, the annular defect was clearly visualized. The needle entry point was carefully chosen in the outer annular tissue, approximately 2–3 mm from the defect margin, where the tissue retained sufficient tensile strength for secure anchorage (Figures 3A,B). Second, in this C6/C7 lesion, the herniation was located superior to the shoulder of the exiting C7 nerve root; therefore, the repair could be performed without significant neural retraction. During the procedure, the suture device itself was used as a gentle retractor to protect the nerve root and dura while guiding needle passage. The first (white) needle was advanced to pierce both medial edges of the annular fissure, forming a traction loop. The second (green) needle was then passed through the white loop and inserted into the lateral edge of the defect (Figure 3C). By pulling on the white loop, the green suture was delivered internally to complete the stitch. Knot tying was performed extracanalicularly using a knot pusher, and the knots were deliberately positioned outside the spinal canal to avoid neural irritation (Figure 3D). Excess suture was trimmed endoscopically (Figure 3E).

Following annular repair, bipolar radiofrequency was applied to coagulate the surrounding tissue and reduce potential irritation from the suture (Figure 3F). Final endoscopic inspection confirmed full decompression of the right C7 nerve root and an intact annular closure with no residual disc material or bone debris. The LF suspension suture was released, allowing the ligament to return to its anatomic position (Figure 3G). The incision (∼1.5 cm) was closed in layers, and a closed-suction drain was removed 28 h postoperatively (Figure 3H). The total operative time was 50 min.

## 4 Postoperative course and follow-up

**Figure 4:**
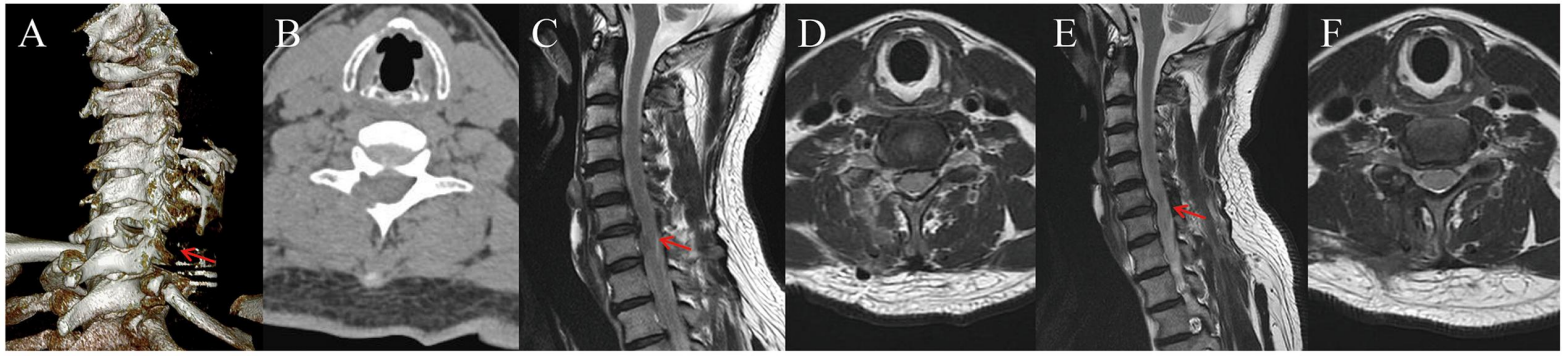
Postoperative and follow-up imaging findings. **(A)** Three-dimensional CT reconstruction on postoperative day 3 demonstrating adequate laminotomy and bony decompression at the C6/C7 level (arrow); **(B)** axial CT image confirming complete canal clearance without hematoma, bone fragment, or malalignment; **(C)** sagittal T2-weighted MRI on postoperative day 3 showing full decompression of the right C7 nerve root and an intact annular closure with mild localized hyperintensity at the suture site (arrow); **(D)** axial T2-weighted MRI depicting resolved lateral recess compression and absence of residual disc material; **(E)** three-month follow-up sagittal MRI demonstrating sustained neural decompression and preserved annular contour (arrow); **(F)** axial follow-up MRI confirming a healed annular repair site with no evidence of recurrent herniation or epidural fluid collection.

Postoperatively, the patient experienced rapid and significant relief of right upper extremity radicular symptoms. Over 24 h, the surgical drain collected approximately 20 mL of blood-tinged fluid. To reduce postoperative nerve edema and reperfusion-related inflammation, intravenous methylprednisolone (80 mg/day) was administered for three consecutive days. No neurological deterioration, wound complications, or dural irritation occurred during hospitalization. On postoperative day 3, cervical MRI and CT were performed to evaluate decompression and annular repair. CT confirmed effective bony decompression at C6/C7 without hematoma or alignment issues (Figures 4A,B). T2-weighted MRI showed full relief of right C7 nerve root compression and no signs of residual disc, effusion, or reherniation. The repaired annulus appeared well approximated without evident gaps or edema. Mild T2 hyperintensity at the suture site suggested early fibrous healing. The LF remained anatomically preserved, and no dural deformation was observed (Figures 4C,D). At the 3-month outpatient follow-up, cervical spine MRI demonstrated sustained neural decompression with no evidence of recurrent disc herniation. The annular closure site exhibited low T2 signal intensity at the prior defect region, consistent with fibrous healing tissue. The annular contour appeared continuous without visible re-tear or inflammatory changes. No epidural fluid collection or neural compression was observed (Figures 4E,F). These findings suggest successful annular integrity restoration and stabilization. Clinically, the patient experienced complete resolution of numbness and pain in the right arm, with only occasional neck soreness after exertion. Functional reassessment demonstrated marked improvements in the VAS score from 7 to 1, the JOA score from 13/17 to 16/17, and the NDI from 42% to 14%, indicating significant neurological and functional recovery in neurological and functional domains.

## 5 Discussion

This case highlights the successful application of the UNSES technique for treating unilateral cervical radiculopathy at the C6/C7 level. Through a minimally invasive posterior approach, precise nerve root decompression was achieved while preserving the LF and maintaining spinal stability. Critically, this procedure incorporated endoscopic annular suture repair—an approach not previously reported in the cervical spine. The integration of targeted decompression with annular closure demonstrates the feasibility of motion-preserving cervical interventions and expands the surgical potential of endoscopic spinal techniques.

ACDF has long been considered the gold standard for managing cervical radiculopathy due to its high success rate and direct decompression of ventral pathology (10). However, fusion inherently reduces cervical mobility and has been linked to accelerated ASD in up to 25% of cases (11). In contrast, PCF, preserves motion segments and avoids fusion-related complications. while endoscopic variants of PCF have demonstrated comparable clinical efficacy to ACDF while offering advantages such as reduced blood loss, shorter hospital stay, and faster return to work (12). Beyond these approaches, conventional posterior endoscopic cervical discectomy (PECD) has been validated as a safe, minimally invasive alternative, achieving excellent neural decompression with low complication rates (13). Reported operative times for uniportal PECD typically range from 60 to 100 min, depending on the level and pathology, with minimal blood loss and a complication rate below 5% (14). Although large-scale data on operative times for cervical UNSES are not yet available, our single-level procedure involving discectomy, decompression, ligamentum flavum suspension, and annular suture repair required approximately 60 min in total. Within this duration, the combined steps of LF suspension and annular closure accounted for about 10 min of operative time. Thus, the overall duration is comparable to that of conventional PECD, rather than significantly longer. Importantly, unlike traditional PECD—which often leaves the annular defect unrepaired and necessitates partial resection of the LF for exposure, potentially predisposing to postoperative epidural fibrosis and disc reherniation—UNSES integrates both temporary LF suspension and direct annular repair, enabling clear visualization, preservation of the posterior tension band, and restoration of annular integrity. Previous lumbar endoscopic studies have shown that annular suturing can significantly reduce recurrence rates and promote fibrous healing (8), while LF preservation minimizes epidural scarring and neural adhesion (9). Therefore, incorporating these steps into cervical endoscopy may improve biomechanical integrity and long-term outcomes. Based on the above considerations, the posterior approach was chosen because the disc herniation was unilateral and clearly localized to the right C6/C7 level, dynamic flexion-extension radiographs demonstrated preserved segmental stability, and the patient expressed a strong preference for a motion-preserving, non-fusion technique. UNSES provided a precise and minimally invasive solution well suited to these clinical and patient-specific indications.

UNSES is a novel endoscopic platform first introduced by Professor En Song in 2021 (15). The technique features a single working portal combined with a noncoaxial design, enabling independent camera and instrument trajectories. This design also allows the endoscope to rotate independently, offering enhanced flexibility and visualization, particularly in complex anatomical corridors. Initially developed for lumbar pathologies, Uniportal Noncoaxial Spinal Endoscopic Surgery (UNSES) has shown clinical efficacy across a spectrum of degenerative and structural spinal conditions. In lumbar disc herniation, UNSES enabled effective decompression with minimal iatrogenic trauma and favorable recovery, including in patients with comorbidities such as hemophilia (15). For extreme lateral disc herniations and foraminal stenosis, the noncoaxial approach allows for safer and more comprehensive decompression within confined spaces (16). Furthermore, its utility has been demonstrated in complex decompressive scenarios such as unilateral access for bilateral decompression (17), and in managing gas-containing lumbar disc cysts using combined posterior and extraforaminal approaches (18). In trauma-related settings, UNSES has been applied to burst fractures requiring decompression and stabilization, with successful symptom relief and preservation of posterior anatomical structures (19). Beyond spinal pathology, this technique has also been adapted for the treatment of benign skeletal lesions such as fibrous dysplasia of the femur, underscoring its potential for minimally invasive management in non-spinal orthopedic contexts (20). Collectively, these reports highlight the growing adaptability of UNSES as a safe, versatile platform across a range of indications. In cervical spine applications, this configuration facilitates precise neural decompression with minimal dural or root manipulation, as instruments can approach from multiple angles without compromising the viewing axis. The use of a 30 ° oblique-view endoscope further augments the field of view, enabling clear identification of critical structures such as the exiting nerve root, foraminal roof, and annular fissure (21). Moreover, the working portal replicates the tactile and visual experience of open surgery in a minimally invasive format, thereby reducing the technical learning curve for experienced spine surgeons transitioning to endoscopy (15). From a safety standpoint, UNSES allows targeted resection of hypertrophic bone and herniated disc material while preserving stabilizing structures such as the facet joint and LF. This contributes to reduced intraoperative bleeding, shortened operative time, and accelerated postoperative recovery (16). In our case, the approach enabled controlled decompression of the right C7 nerve root with full visualization and successful annular repair, achieved through a minimal laminotomy and muscle-sparing access. Notably, the flexible instrument trajectory and clear visualization provided by the UNSES configuration were pivotal in enabling precise annular closure within the limited cervical operative corridor. Compared to biportal systems, the uniportal setup ensures alignment between the operative field and endoscopic view, allowing clear assessment of both needle entry and exit points during suturing, thereby minimizing the risk of neurovascular injury. Moreover, the use of a single working channel allows both suture limbs to be manipulated in the same space, simplifying knot tying, avoiding entanglement, and facilitating efficient soft tissue management during anchoring. Additionally, the noncoaxial nature of UNSES provides greater maneuverability compared to coaxial endoscopy, allowing effective exploration and repair of large annular defects that might otherwise exceed the reach of traditional coaxial instruments.

The integrity of the annulus fibrosus plays a critical role in maintaining intervertebral disc biomechanics and preventing recurrence following discectomy. Although cervical annular repair has been rarely reported in clinical practice, existing lumbar spine literature underscores its importance. Studies have shown that wider annular defects are strongly associated with higher recurrence rates, particularly in patients undergoing non-fusion procedures (22). Furthermore, disruption of the annular continuity may predispose to progressive segmental degeneration (23). In this case, we adopted a two-step annular closure using a spinal endoscopic suture device (2020 Inc., China). The first needle established a guiding suture loop on the medial aspect of the annular fissure, followed by the second stitch on the lateral margin. Knot tying was carefully performed to avoid neural contact, ensuring a tensioned closure that approximated the disrupted annular margins. This approach resulted in no visible gap or residual disc material on postoperative MRI. Prior work with annular suture in the lumbar spine has demonstrated favorable remodeling responses, reduced epidural effusion, and lower reherniation rates (24). Before performing annular closure, meticulous hemostasis is essential to maintain a clear endoscopic view and to allow accurate identification of annular margins. The texture and integrity of the annular tissue should be carefully assessed to ensure it is sufficiently firm to hold sutures. The entry and exit points of the suture path must be strategically planned according to the annular tear configuration. Excessive or repeated needle passes should be avoided, as they may further compromise annular strength and accelerate degenerative changes. To our knowledge, this is the first clinical application of endoscopic annular closure in a cervical discectomy setting. While technical challenges exist due to the confined cervical anatomy, the combination of high-definition noncoaxial endoscopy and minimal neural retraction enabled safe and precise repair. Our group has previously reported successful annular suturing under endoscopy in lumbar cases with excellent outcomes and this technique appears transferable to cervical applications with proper patient selection and operative expertise (15, 24).

Preservation of the LF during spinal decompression has been increasingly recognized as a valuable strategy to minimize postoperative epidural fibrosis, dural adhesions, and iatrogenic instability (25). Beyond its protective barrier function, the LF plays an essential biomechanical role as part of the posterior tension band, contributing to elastic recoil during spinal extension and stabilizing interlaminar motion. Biomechanical studies have shown that disruption of the LF can alter the posterior load-sharing mechanism, increasing intradiscal pressure and facet joint stress, thereby predisposing to postoperative instability and accelerated degeneration (9). While most of the existing evidence comes from lumbar spine research, the underlying biomechanical and protective principles remain highly relevant in cervical applications. Özay et al. reported that LF-sparing lumbar microdiscectomy significantly reduced epidural scarring and improved pain scores (26). Similarly, Aydın et al. found that patients undergoing LF-preserving surgery had lower recurrence rates (1.75% vs. 4.5%) and fewer reoperations (4.5% vs. 9%) compared to those receiving conventional discectomy (27). In the cervical spine, Ishikawa et al. demonstrated that preservation of posterior elements, including the LF, may contribute to improved neurological outcomes in patients with coexisting ossifications such as ossification of the posterior longitudinal ligament (OPLL) and OLF (28). Building on this evidence, our approach adopted the principles of structural preservation through a minimally invasive interlaminar corridor. The LF was gently suspended using 4-0 absorbable sutures, placed percutaneously and anchored externally. The use of fine absorbable sutures minimizes shear or cutting injury to the ligament while providing sufficient tensile strength to support the suspension force, which in practice is approximately equivalent to the weight of a straight surgical clamp. This gentle elevation of the LF effectively exposes the spinal canal and operative field without compromising the integrity of the preserved tissue. It should be noted, however, that in patients with markedly hypertrophied or fibrotic LF, this preservation technique may not be suitable due to limited elasticity and reduced handling safety. Finite element and cadaveric studies indicate that the LF remains within its physiological elastic range under tensile loads of approximately 1 N, well below the threshold for structural damage (29).

Although UNSES provides an anatomically preserving and minimally invasive alternative for selected cases of cervical radiculopathy, several limitations should be acknowledged. Cervical endoscopic annular suturing is technically demanding due to the narrow workspace and proximity to neural structures. It requires advanced endoscopic proficiency and should not be attempted by beginners. Surgeons experienced in lumbar endoscopic discectomy and annular repair are better suited for this procedure (13). Nevertheless, this technique is not universally applicable to all cervical disc herniations. It is most appropriate for soft, paracentral herniations such as the present C6/C7 case, where neural tension is moderate and the annular defect can be safely accessed for suturing. Central or calcified herniations, upper-level lesions (C4–C5 or above), or cases with segmental instability or OPLL may not provide sufficient space or safety margins for annular repair. These limitations align with previous findings that cervical endoscopic surgery is best suited for lateralized, non-calcified lesions without significant spinal cord compression (30). Future multicenter studies with larger patient cohorts are warranted to refine the learning curve, optimize training standards, and better define the selection criteria for safe and effective cervical endoscopic annular repair.

## 6 Conclusion

In summary, UNSES enabled minimally invasive posterior cervical discectomy with successful annular repair and LF preservation. As the first reported case of cervical annular repair under endoscopy, this technique shows promise as a motion-preserving alternative for cervical pathology.

## Data Availability

The original contributions presented in the study are included in the article/Supplementary Material, further inquiries can be directed to the corresponding author.
